# Including refugees in disease elimination: challenges observed from a sleeping sickness programme in Uganda

**DOI:** 10.1186/s13031-017-0125-x

**Published:** 2017-12-01

**Authors:** Jennifer J. Palmer, Okello Robert, Freddie Kansiime

**Affiliations:** 1 Centre of African Studies, School of Social and Political Sciences, University of Edinburgh, 15a George Square, Edinburgh, EH8 9LD UK; 2 Health in Humanitarian Crises Centre, London School of Hygiene & Tropical Medicine, Keppel Street, London, WC1E 7HT UK; 3 London School of Hygiene & Tropical Medicine, Keppel Street, London, WC1E 7HT UK; 4 Department of Public and Community Health, Busitema University, PO Box 236, Tororo, Uganda

**Keywords:** Sleeping sickness, Human African trypanosomiasis, Refugees, Uganda, South Sudan, Elimination, Integration, Ethnography, Displacement, Rapid diagnostic tests

## Abstract

**Background:**

Ensuring equity between forcibly-displaced and host area populations is a key challenge for global elimination programmes. We studied Uganda’s response to the recent refugee influx from South Sudan to identify key governance and operational lessons for national sleeping sickness programmes working with displaced populations today. A refugee policy which favours integration of primary healthcare services for refugee and host populations and the availability of rapid diagnostic tests (RDTs) to detect sleeping sickness at this health system level makes Uganda well-placed to include refugees in sleeping sickness surveillance.

**Methods:**

Using ethnographic observations of coordination meetings, review of programme data, interviews with sleeping sickness and refugee authorities and group discussions with health staff and refugees (2013–2016), we nevertheless identified some key challenges to equitably integrating refugees into government sleeping sickness surveillance.

**Results:**

Despite fears that refugees were at risk of disease and posed a threat to elimination, six months into the response, programme coordinators progressed to a sentinel surveillance strategy in districts hosting the highest concentrations of refugees. This meant that RDTs, the programme’s primary surveillance tool, were removed from most refugee-serving facilities, exacerbating existing inequitable access to surveillance and leading refugees to claim that their access to sleeping sickness tests had been better in South Sudan. This was not intentionally done to exclude refugees from care, rather, four key governance challenges made it difficult for the programme to recognise and correct inequities affecting refugees: (a) perceived donor pressure to reduce the sleeping sickness programme’s scope without clear international elimination guidance on surveillance quality; (b) a problematic history of programme relations with refugee-hosting districts which strained supervision of surveillance quality; (c) difficulties that government health workers faced to produce good quality surveillance in a crisis; and (d) reluctant engagement between the sleeping sickness programme and humanitarian structures.

**Conclusions:**

Despite progressive policy intentions, several entrenched governance norms and practices worked against integration of refugees into the national sleeping sickness surveillance system. Elimination programmes which marginalise forced migrants risk unwittingly contributing to disease spread and reinforce social inequities, so new norms urgently need to be established at local, national and international levels.

**Electronic supplementary material:**

The online version of this article (10.1186/s13031-017-0125-x) contains supplementary material, which is available to authorized users.

## Background

The Sustainable Development Goals (SDGs) encourage states not to ‘leave behind’ populations who have been forcibly displaced by war and other extreme hardships in development work. Such forced migrants include people who are internally displaced to areas within their own country where different languages, ethnic groups and customs may predominate, as well as refugees who have left their country and are seeking protection from another.

Finding effective ways to include forcibly-displaced populations in global elimination programmes is important as conflict-affected regions are often the places where disease is most intractable [1–4]. It is particularly important for elimination of sleeping sickness (a fatal parasitic infection also known as human African trypanosomiasis or HAT) as outbreaks in the past have been associated with forced migrations [5–8]. Populations migrating to avoid conflict or returning after displacement are particularly vulnerable to sleeping sickness through: exposure to tsetse flies, which carry the disease, when settling rural uninhabitated areas; famine and stress which may make infected carriers more likely to develop disease and transmit infection; as well as difficulty accessing health services to detect and treat the disease [5, 9, 10]. Syndromic-based detection of sleeping sickness during routine care visits, which requires health staff to recognise symptoms variably affecting the mind and several body systems and producing different meanings in biomedical and customary health systems, may be particularly difficult in a cross-cultural context [5, 11]. Humanitarian agencies such as Médecins Sans Frontières (MSF) who have been key providers of sleeping sickness services for conflict-affected populations in the past are disengaging from control as disease prevalence declines. It thus increasingly falls to national programmes and partnerships to secure displaced peoples’ inclusion in elimination activities. Here, we report on governance challenges experienced by Uganda’s sleeping sickness elimination programme to include South Sudanese refugees in facility-based medical surveillance.

Two recent promising, but relatively unstudied, global policy trends provide favourable conditions for ensuring refugees’ access to sleeping sickness surveillance in national elimination programmes: the development of rapid diagnostic tests (RDTs) for use in frontline facilities and the adoption of refugee policies which integrate health services for refugees in national systems.

With sleeping sickness infection recognised as both an outcome and driver of poverty, programme outcomes for this and other ‘neglected tropical diseases’ (NTDs) have been proposed as tracer indicators for a number of other SDG targets to monitor social equity [12]. Similarly, global NTD plans stress the need to make disease control services universally accessible at the primary healthcare level [13]. For sleeping sickness, this has become more realistic within the last few years with the development of RDTs which, unlike previous diagnostics, do not require electricity, refrigeration or specialised technical expertise to administer, although further parasitological confirmation is still required before treatment can be given. Because RDTs can be integrated into the routine activities of primary healthcare facilities, the need for external actors to support expensive, independent mobile teams who screen at-risk populations systematically is theoretically less important. This new technology therefore enables a shift in sleeping sickness control governance away from a largely vertical approach, often with multiple actors working in parallel on short-term objectives, towards a long-term, coordinated approach appropriate for elimination that is integrated into public health systems and strengthens them [5, 14]. The use of RDTs to secure access to sleeping sickness services for forced migrants, however, may involve additional social and governance-related considerations given that forced migrants typically face numerous constraints to health, including the embodiment of social stresses related to their experience of exclusion or marginalisation [15–17]. Refugee health and agency is particularly influenced by the policies of humanitarian and receiving country government systems.

The idea that refugees should be integrated into national development projects has been proposed as a policy solution to the negative effects of social marginalisation on refugee health since the 1980s [18]. Governmental, humanitarian and development actors should arguably share responsibility for displacement because promoting long-term refugee well-being and independence from aid is also good for the host community. Host communities typically face the same regional development challenges as refugees, such as inadequate healthcare. Addressing refugee needs sustainably can therefore benefit everyone “like a rising tide lifts all boats” [19]. While ‘interim integration’ of some services such as for healthcare is increasingly popular [20], most African states typically oppose comprehensive social integration which uniformly grants refugees the same rights as host nationals, including to claim citizenship or permanent residence [18]. Within the field of public health, there has been remarkably little reflection on the implications of different refugee healthcare governance models on long term goals such as disease elimination [20].

The current humanitarian crisis in South Sudan has caused the displacement of more than 3.7 million people, including 1 million refugees to Uganda since December 2013 [21], particularly to the north-western West Nile Region where the Ugandan government is operating a programme to eliminate sleeping sickness. This area was the first in Africa to integrate sleeping sickness RDTs into primary healthcare facilities on a large scale. Uganda has also pioneered a refugee policy which favours integration of primary healthcare services for refugee and host populations [22], making the region well-placed to incorporate refugees into sleeping sickness surveillance activities. Nevertheless, one year into the response, an incongruous situation emerged in which sleeping sickness RDTs, a key surveillance tool and indicator of access to sleeping sickness care, had been removed from facilities serving high concentrations of refugees who were believed to be at risk for the disease. This exacerbated an already existing gap in equitable access to elimination initiatives between host and refugee populations in West Nile that persisted for at least 3 years. Through close examination of the politics and experiences of refugees and implementers, this study investigated issues with these tandem processes of integration of technologies and people into government systems to explain this inequitable outcome and understand key governance challenges sleeping sickness programmes may face to achieve SDG equity goals among populations of forced migrants.

## Methods

### Elimination context in West Nile

Successive forced migrations over the South Sudan-Ugandan border have been associated with successive epidemics of *gambiense*-type sleeping sickness in both countries since at least the 1970s [7–9, 23, 24]. The most recent epidemic was associated with the Central and East African wars of the 1990s. Uganda reported a peak of 1123 cases in 1997 and South Sudan a peak of 3121 in 2002 [5]. Measures by government, humanitarian agencies, research organisations and coordinating bodies such as the Uganda Trypanosomiasis Control Council (UTCC) and its secretariat, the Coordinating Office for Control of Trypanosomiasis in Uganda (COCTU), helped achieve a steady reduction of cases in both countries since the 1990s, despite continued large-scale conflict and post-conflict return migrations. In 2013, only 117 cases were reported from South Sudan and only 9 from Uganda [25].

The elimination of *gambiense* sleeping sickness was declared feasible at a global level in 2011 [26], but, in practical terms, this was not true for Uganda or South Sudan until they entered partnerships with the Foundation for Innovative New Diagnostics (FIND) to introduce new diagnostics, including RDTs, to strengthen their health systems and “accelerate” progress towards elimination [27]. The low case load in both places had deterred humanitarian actors from maintaining their outreach-based sleeping sickness programmes. The national programmes became reliant on a passive approach to case detection, with screening services for the West Nile Region’s 2.2 million people, for example, only available at four facilities. While Uganda was deemed capable of maintaining disease control at this prevalence without external support, this approach to case detection was not thought adequate to achieve elimination [14].

**Fig. 1:**
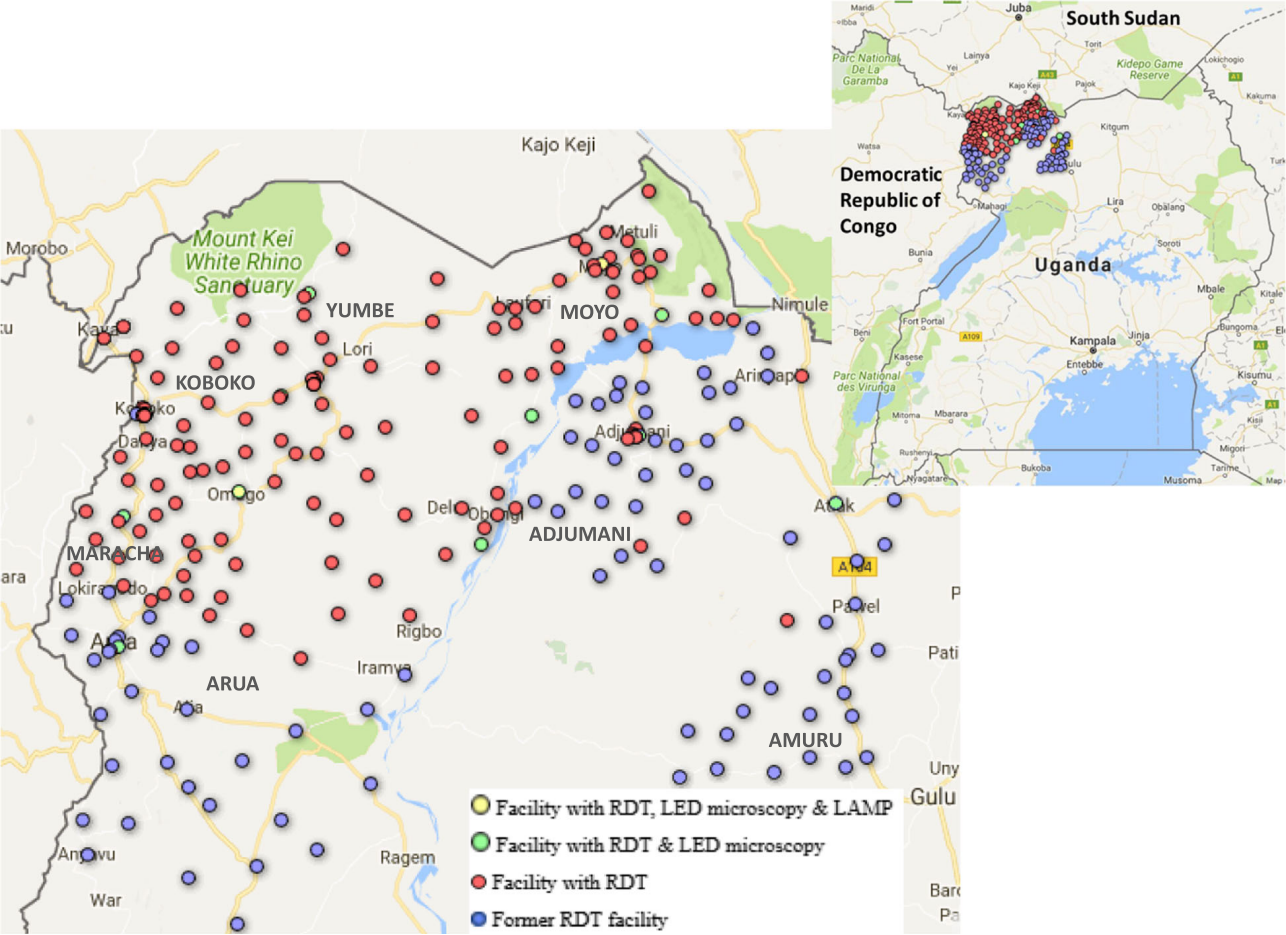
Map of West Nile region in north-west Uganda showing locations of health facilities with sleeping sickness diagnostic capacity. Legend: Facilities using sleeping sickness RDTs are represented as red circles, facilities where RDTs have been withdrawn are shown in blue, facilities with HAT LED microscopy capacity in green and those with LAMP capacity in yellow. District names within West Nile are shown in block capitals. See [54] for original, interactive online map to access more information about timing related to RDT introduction, withdrawal (and in some places, re-introduction) in individual facilities. In August 2014, the only facilities with RDT-based surveillance capability were Adjumani Hospital and the level 4 health centre in Mungulla

Under the FIND-supported and multi-donor-funded Intensified Sleeping Sickness Elimination Programme (ISSEP), the Ugandan Ministry of Health has distributed sleeping sickness RDTs to over 200 frontline facilities and hospitals since mid-2013 (Fig. 1 and Additional file 1) [27].^1^ These covered the limits of *G fuscipes*-type tsetse habitat which supports *gambiense*-type sleeping sickness in the country. A similar programme was initiated in areas of South Sudan abutting Uganda in 2015, although insecurity since 2016 means many activities have been suspended. Distribution of RDTs was accompanied by a one-day workshop for staff in each facility to train them in how to recognise symptoms of the disease and use the RDT on syndromic suspects they came across in their routine work. With HAT prevalence at such a low level in this setting, fewer than 1 of every 100 patients who test positive with the RDT is expected to be a true case [28]. Twelve referral facilities were thus also equipped with fluorescent LED microscopes and three with LAMP machines to confirm patients screened with RDTs. Under this strategy, 19 cases had been identified as of September 2017, including three among South Sudanese refugees and migrants (two in 2017).

### Refugee health policy context in Uganda

By global standards, Uganda has an inclusive refugee policy which recognises refugees’ right to health, education, work and move freely. International health responses for refugees have been actively integrated into local government-managed systems since 2000 [19]. Exclusionary practices in other sectors, however, are believed to have harmful effects on refugee health and well-being [29].

Refugee studies scholars routinely criticise the government- and the United Nations Refugee Agency (UNHCR)-led practice of driving most refugees into settlements [29–32], a type of open camp setting managed by the Office of the Prime Minister (OPM) and humanitarian agencies in sparsely populated rural areas loaned from local communities. Importantly, settlements segregate refugees from the local population by allocating refugees land that is neither adequate for nutritional self-sufficiency nor close enough to urban centres to enable other forms of livelihood and access to cash [30]. Despite sometimes violent conflicts with the host community over resource use [31, 32], neither police nor development actors tend to engage with refugees, viewing settlements as temporary phenomena [29, 32]. Settlements can thus ultimately make refugees vulnerable both socially and economically, and reinforce difference [30].

The settlement system can also sometimes work against refugee integration into the health system. As humanitarian resources need to be seen as benefiting both host and refugee communities, health funding raised by UNHCR is targeted to existing government primary healthcare structures near refugee settlements, either through direct contributions to the OPM which disburses it to districts which manage the facilities, or to non-governmental organisations (NGOs) to expand services at them. Refugees self-settling outside of official settlements benefit from no specific humanitarian health interventions. Very few additional resources are directed to secondary and tertiary facilities which are also inaccessible when people in settlements cannot pay for taxis as ambulances are scarce. Vertically-organised government programmes which require the coordination of resources beyond primary care are implicitly expected to absorb refugee needs. Recent failures among these programmes have included high profile epidemics of malaria [33, 34], hepatitis B [35] and malnutrition [33] whereas an outbreak of measles was largely avoided because of pre-emptive humanitarian-led vaccination [33]. While in popular media refugees have long taken the blame for such outbreaks in Uganda [34–36], infectious disease threats from host communities to refugees are seldom recognised politically [34]. Integration of healthcare services is historically perceived as having improved healthcare for host nationals in Uganda but worsened it for refugees [20]. Today, refugees living both inside and outside settlements continue to characterise access to health services as inadequate [30].

### Research approach

To understand if displaced populations are being left behind in development programmes, refugee studies scholars suggest examining whether displaced and hosting area populations are equally able to access the same resources, including whether similar per capita amounts are provided for each population, adjusted for disease prevalence [20]. In the context of sleeping sickness elimination, we considered a marker of ‘access’ to be whether RDTs were used at similar rates in refugee and host populations, given that screening with RDTs is a precursor to all further case detection and treatment action by a programme.

As the ISSEP had not set-out to prospectively monitor access equity, we used available facility-based passive screening data from the programme (calculating the number of RDTs used per facility and per endemic district over time) and compared this to UNHCR data on the sizes of refugee populations hosted across districts to discern, in broad terms, possible disparities in surveillance access for refugees. We also attempted to explore refugees’ own perceptions of their access to sleeping sickness surveillance, including how this contributes to their social integration and therefore good health [20]. To explain the disparities that we identified, we employed a variety of qualitative methods to understand evolving operational and governance challenges. We paid particular attention to the processes through which policies, norms, power and language influence decision-making within the ISSEP (across West Nile from December 2013 to September 2016) and within humanitarian health responses (predominantly in Adjumani District, from July 2015, Table 1).

**Table 1:** Summary of research methods used according to integration process studied

Research methods used	Integration of sleeping sickness case detection into general health services	Integration of refugee responses into general health services
Ethnographic observations during meetings	2013–2016: Observations of discussions about ISSEP at 6 high level national and international meetings and conversations with discussants; 2013: Observation of 4 ISSEP training days and conversations with trainers	
Interviews with key informants	2013–2015: Repeated interviews with 8 field supervisors in all districts	2015: Interviews with representatives of 4 government and non-governmental authorities coordinating refugee health responses and clinical staff at 4 health facilities serving refugees
Focus group discussions (FGDs)	2013: 4 FGDs held with 24 health workers (mixed genders) undergoing ISSEP training in Arua, Yumbe and Moyo Districts; 2015: 6 FGDs held with 47 refugees in Adjumani District settlements (3 Dinka groups, 3 Madi, mixed genders, ≥18 years old)	2015: 6 FGDs with refugees (see last column)
Review of programme data	Review of RDT monitoring data shared by ISSEP	Review of population data shared by UNHCR

Research activities were conducted alongside and drew on material generated for smaller research studies commissioned by the ISSEP for programme improvement and written up in technical reports [37–39]. Information on historical and contemporary approaches to sleeping sickness service governance and integration in West Nile came from: ethnographic observations and conversations with people participating in high level sleeping sickness coordination meetings, observation of ISSEP training activities and focus group discussions (FGDs) with health workers undergoing the training and interviews with ISSEP field supervisors. Key topics investigated and analysed were: expectations about how RDTs should be deployed and used by workers within the general health system, decision-making surrounding emerging challenges, and plans to control sleeping sickness in migrant and/or refugee populations. FGDs with health workers (labelled in the analysis as ‘*District* training FGD’ with ‘District’ referring to where they occurred) as well as all interviews and meetings were conducted in English. FGDs were recorded and transcribed, as were ethnographic interactions when possible, otherwise field notes with verbatim quotations were made on the spot and expanded on later in the day.

Information on the integrated organisation of care for refugees came from: interviews conducted with representatives of government and non-governmental authorities as well as staff at health facilities serving refugees, which were recorded and transcribed. Challenges organising and delivering care were explored, including how humanitarians accounted for vertical programming needs, particularly the need for sleeping sickness control.

We collected information from refugees on their experience of both processes of integration using FGDs, covering the following topics: typical health problems refugees face, experiences of healthcare in Uganda and elsewhere, knowledge and experience of sleeping sickness, awareness of sleeping sickness RDT availability and suggestions for how to improve services for refugees. FGDs were conducted in local languages by research assistants recruited from refugee settlements and trained using methods described in [40]. Participants were recruited through natural groupings such as women’s or men’s groups and excluded anyone who worked at a health facility. Material from each FGD was discussed by the research team during immediate de-briefings as well as after full translated transcripts were produced to clarify details and check translated phrasing.

We selected refugee participants by targeting settlements we believed had the highest chance of containing people exposed to sleeping sickness (see Additional file 1 for further detail). As of July 2015, Nimule, which has hosted indigenous ethnic Madi and internally displaced Dinka populations over the last decade, appeared to be the sleeping sickness-endemic area within South Sudan subject to the most forced migration. FGDs were thus conducted in the following settlements which contained people who lived in or migrated through Nimule, all of which were located in Adjumani District: Maaji 1 (labelled in the analysis as Madi FGD A), Maaji 2 (Madi B and C), Ayilo 1 (Dinka A and B) and Nyumanzi (Dinka C).^2^^,^ 3

Transcripts and notes from interviews, FGDs and observations from both research strands were analysed thematically using NVivo software and combined in the final analysis to identify the limitations of each integration strategy and how they interacted to unintentionally limit refugees’ access to sleeping sickness surveillance. Some excerpts from FGD and interview transcripts presented below have been edited for clarity, while attempting to preserve the tone and meaning of the original translation. All contributions have been anonymized.

## Results

### A gap in surveillance equity

In 2014, a year into the programme, Adjumani District, which hosted the largest refugee population (87% of refugees in the region), had used the least amount of RDTs and had the lowest rate of RDT use (1.2 per facility per month on average in Adjumani District versus 3.9 in the region, Table 2), suggesting inequitable access to surveillance for the majority of refugees living in West Nile. This inequity was exacerbated when, at the end of that year, managers decided to withdraw RDTs from facilities in three districts: Amuru, Adjumani and southern Arua, the latter two the only places hosting refugees. In Adjumani, whereas RDTs had initially been deployed in 36 facilities, RDTs remained available in just two hospitals (Mungulla Health Centre 4 and Adjumani District Hospital, Fig. 1 and Table 2). One was located near a refugee settlement but most refugees lived more than an hour’s drive away from either facility.

**Table 2:** Refugee population compared to rate of facility RDT use by district, Aug 2013-Jun 2014

District	Refugee population (Jun 2014)^a^	# RDTs used per facility-month (Aug 2013 – Jun 2014)	# Facilities using RDTs	# Facility-months RDTs available	# RDTs used
Koboko	–	9.5	14	135	1279
Maracha	–	5.3	14	140	742
Yumbe	–	5.4	24	165	883
Arua	11,098	3.4	57	465	1563
Moyo	–	2.8	40	218	608
Amuru	–	2.5	27	135	337
Adjumani	76,043	1.2	36	176	217

^a^ Population data refers to South Sudanese refugees who came to Uganda after 15 Dec 2013 and had registered with UNHCR by 17 Jun 2014 [55]. No settlements hosting South Sudanese refugees existed outside of Adjumani and Arua Districts at this time. RDT data was shared by the programme and analysed in [38]

In recognition of the need to reach refugees better as the crisis grew, the ISSEP began to re-introduce RDTs into some Adjumani facilities in 2016 and conduct active screening in some settlements. This 2013–16 period however, represents a likely example of inequitable access for refugees to sleeping sickness surveillance and control in West Nile, the effects of which we describe from the perspective of settlement populations next.

#### Refugee perspectives on surveillance access

When we spoke with refugees in Adjumani settlements in 2015, the limitations of access were clearly felt. Only one Maaji settlement resident mentioned knowing that sleeping sickness tests were available in Adjumani town. Otherwise, people in all group discussions stated they did not know how they could be tested, despite clear concern about their risk of disease.

Participants knew about sleeping sickness transmission as well as the major signs and symptoms from past experiences with the disease. Madi participants were particularly concerned about their risk of sleeping sickness from the environment. As Maaji residents put it, “there are too many flies, as this place was only for animals” (participant 3, Madi FGD C), referring to the idea that their settlements bordered the Zoka game reserve which had been abandoned by local people during the Ugandan war with the Lord’s Resistance Army. Until UNHCR developed the land for South Sudanese refugees in 2014, residents said “this place was just bush” (participant 4, Madi FGD A) and not fit for human habitation. This worried people because bushy areas they had lived in during previous displacements had caused them to get sleeping sickness:

“the disease affected my son during the first war era at border areas […he had] bad dreams and he was always isolated, not playing with friends […] As a babysitter, he would go […] with the parent of the child to the garden which is deep in the bush, so he got it from there” (participant 1, Madi FGD A)

While Dinka participants in Nyumanzi and Ayilo settlements said they did not get bitten by tsetse in their current settlements, fear or dislike of bushy areas was a key reason that Dinka people in Nyumanzi protested being moved to a new settlement in Yumbe in 2016 [41].

Residents in all settlements worried there could be people living among them or in neighbouring host communities who were spreading sleeping sickness. Participants therefore requested access to screening services because people believed cases “are there but we do not know them” (participant 2, Dinka FGD B) and “patients with the disease are not allowed to mix with other people in the settlement” (participant 4, Madi FGD A). Residents also wanted access to ensure that they themselves weren’t infected, suggesting health agencies “should come here in the field with tools to test the entire community so that everyone knows his or her status” (participant 7, Madi FGD A). Participants advocated services be extended to refugee areas in particular, arguing that humanitarian agencies had consistently emphasised in the past that sleeping sickness was of special concern to displaced populations: “They [humanitarian agencies] went to check refugees first […] because we, the refugees, are affected by most diseases” (participant 5, Madi FGD C).

Sharing statements such as: “Screening facilities for sleeping sickness are not here in Uganda but in South Sudan they are common in places like Yei, Maridi, Yambio and Nimule side” (participant 7, Dinka FGD A), refugees therefore unanimously stated that their access to sleeping sickness screening was better in South Sudan, before displacement, than in Uganda. Moreover, in a situation of displacement where “life […] is hard […and] different things can kill you at any time” (participant 4, Madi FGD B), not having control over risk from such a disease appeared to add to refugees’ sense of social exclusion.

### Understanding the unintended equity gap

#### ISSEP outlook towards migration

This equity gap was unintended. From its origins, cross-border migration was perceived as a challenge Uganda’s ISSEP would need to address and this perception increased in urgency as the conflict in South Sudan continued to grow [42]. At RDT training workshops in 2013 before the refugee influx, for example, coordinators asked health providers to “look out” for imported cases from among people migrating for work, healthcare or to visit family, saying, “Cases can spill over […] if our neighbours are not doing their work […]. Be on the look-out for people from [South] Sudan, asking yourself, ‘Are they safe? Are they clean?’” Health workers believed that having RDTs under the ISSEP would assist them to help migrants with sleeping sickness; otherwise, as one health worker argued, “when they come like this, we miss them and we miss the opportunity to diagnose sleeping sickness” (participant 1, Arua training FGD).

Specific concerns about refugee movements surfaced six months later, by which time the conflict in South Sudan had prompted more than 70,000 people to cross the border to refugee settlements in West Nile. At an annual review meeting in May 2014, a member of the government’s UTCC questioned ISSEP coordinators directly, saying: “We have strong hope that Uganda will be able to eliminate but you dampen our spirits when you talk about South Sudan.” A member of COCTU particularly highlighted the risks of refugees living in Uganda’s “hinterland”, unused rural areas which could be expected to have tsetse flies but poor health surveillance, saying: “South Sudan needs to be taken seriously. Refugees who come into the north should not be allowed to go to the hinterlands until they are screened. […] Those found positive should not be allowed to come in.”

Such demonstrable concern for disease transmission from refugees, however, appeared incongruent with the way RDTs as surveillance resources were used within the ISSEP, as already described. Below, we argue that this mismatch of intention and response in practice can be explained by four key governance challenges that made it difficult for the programme to recognise and correct inequities affecting refugees: (a) donor pressure to reduce the ISSEP’s scope in the absence of clear international elimination guidelines on surveillance quality; (b) the local legacy of programme relations with refugee-hosting districts which strained supervision arrangements that should ensure surveillance quality; (c) difficulties that government health workers faced to produce good quality surveillance in a crisis; and (d) reluctant engagement between the ISSEP and humanitarian structures.

#### Pressure to reduce programme scope

For national programme coordinators, a key rationale for integrating the new diagnostic technologies in general health services was to increase health staff participation in surveillance but this was inconsistent with donor needs to contain costs. As intervention staff described in ISSEP training lectures in 2013, many technically capable facilities did not offer the services they potentially could because they had never been supported to use available but cumbersome diagnostic tools. Since RDTs were easy enough to use by any health worker, trainers sought to motivate facility staff to break from “business as usual” and encourage “everyone” to be involved. Technological inclusiveness came at a financial cost, however. In ISSEP coordination meetings programme managers were thus requested by FIND to discuss strategies to best target resources and supervision workload across West Nile [43]. Primarily this involved progressively “dropping” facilities from the programme where deploying surveillance resources was harder to justify, and which FIND estimated to cost around USD 300 each to support in the first year [44].

Additionally, RDTs are such a new technology that international elimination strategy offers unclear guidance on how long they should be deployed, and at what intensity they should be used under an integrated primary health care approach without detecting cases, before an area can be considered disease free [28].^4^ Many areas in West Nile had not reported a single case in the five years prior to the ISSEP, a key metric in global sleeping sickness elimination monitoring [26]. Given that so little systematic mobile team-based screening had been done recently, however, all facilities in areas considered at risk were purposely included in the programme. Coordinators reasoned that if no cases continued to be found after introducing RDTs, these areas could be assumed to have eliminated sleeping sickness and the programme could reduce surveillance to a restricted number of ‘sentinel’ sites. Staff at the ‘dropped’ facilities would continue to refer possible cases to sentinel sites based on syndromic suspicion. If these sites then identified cases, this would alert authorities to continued transmission in the area which could trigger reactive interventions. The programme chose their one year anniversary, August 2014, to withdraw these resources, focussing on facilities at the southern and eastern edges of the endemic zone. As the last place to receive RDTs, however, they had only been in use in Adjumani District for six months. Most facilities serving refugee settlements had only ever used less than 10 RDTs before they were dropped from the programme.

In withdrawing RDTs from Adjumani District, ISSEP decision-makers do not appear to have considered how the programme’s interactions with refugees may have affected the quality of surveillance upon which these decisions were made. Rather, RDTs were withdrawn mainly because they were being used at health facilities at low levels. While low case numbers with high RDT use would suggest the low disease endemicity here expected at the ISSEP design stage, low case numbers with low RDT use suggested to some ISSEP managers a lack of commitment by facilities to effectively implement the intervention which wouldn’t be justifiable to FIND’s donors. As one ISSEP coordinator reasoned in an interview, there was “no point in spending resources on supervision if RDTs were not being used”. Other coordinators who worried surveillance hadn’t been given enough of a chance to find cases here felt their hands were tied by donor expectations, citing long experience of having to implement donor-funded projects with “strings attached” and austere exit strategies.

#### Local supervision legacies

To build sustainable supervision structures, the ISSEP recruited supervisors from a pool of people already employed by districts who would have many additional responsibilities besides sleeping sickness programming. People who were most motivated in previous sleeping sickness programmes were particularly favoured to supervise integration locally. As described in a 2013 interview, ISSEP coordinators believed being selective within this pool was important because successful technological integration depended on supervisors engaging facility staff “to win their hearts”. This also, however, carried a parallel assumption that programme failure could “only be due to the attitude of the health workers”.

In places without a legacy of successful relationships with vertical sleeping sickness projects, such as Adjumani, therefore, the ISSEP coordinators felt they were at a disadvantage. For example, they felt that the following characterisation by an international expert who managed a sleeping sickness programme in 2001–2 was still valid in 2015: “Moyo was always the star focus but disease reduced early on here. Adjumani was always a disaster because we could never interest people in the lab to get involved”. Similarly, when discussing staff problems in another district of West Nile, an ISSEP coordinator in 2014 acknowledged that in the ISSEP design stage, they thought, “if there was a way of leaving them out, we would have. People have been very difficult there, but they’d be an island” in a programme aiming for regional elimination.

Other unattractive characteristics of Adjumani District, such as its extreme heat and poor accommodation options for coordination staff, meant that it was usually treated as an outpost of neighbouring Moyo district. Partly this reflected Adjumani’s relatively new district status. It had been carved out of greater Moyo district in 2006 and COCTU struggled to interest the new authorities in trypanosomiasis control. Thus, despite having a similar number of participating facilities as other districts (Table 2), for the first half of the programme Adjumani never had its own supervisor, borrowing supervision resources instead from Moyo. This effectively halved the district’s “in-person supervision” time stressed during ISSEP training events as so important for encouraging RDT use and identifying implementation problems which could affect surveillance data quality. What would become Uganda’s biggest refugee hosting district in the region was therefore never expected to integrate sleeping sickness detection technologies well but was also arguably never supported sufficiently to do so.

#### Difficulties using RDTs in a crisis

Almost immediately there appeared to be implementation challenges in Adjumani facilities serving refugee populations affecting both intra-facility dynamics among staff and dynamics between providers and patients. With Nyumanzi Health Centre staff increasing from 2 people before the South Sudan conflict to more than 30 by 2015, for example, entirely new management structures were introduced. Given that staff who had originally been trained to use RDTs by the ISSEP typically occupied the lowest cadres employed in the new structures, this sometimes made sharing knowledge and norms about RDTs introduced by the ISSEP to other staff awkward.

Patient-provider communication necessary to prompt decisions to use sleeping sickness RDTs was also described as very difficult by both staff and refugees. Translators, for example, were scarce at the hospitals which would become sentinel surveillance sites. As described by a senior medical staff member at Adjumani District Hospital: “they are very few so they cannot be everywhere, like in the OPD [outpatient department] we do not have”. But even at lower-level facilities where translation assistance was more available, Dinka people feared they were often misunderstood with the consequence that, “you may not be treated, or if you get an improper translation you get the wrong medicine” (participant 2, Dinka FGD C). Even Madi-speaking refugees living in a Madi-speaking area of Uganda complained of similar communication problems at facilities staffed by non-Madi Ugandans recruited for the humanitarian surge response, as in the following description:

“the translator laughed at me and told me I should return with my husband. So I questioned him, ‘why should I go with my husband and what was the problem?’ He just told me I should understand what he is telling me. The second time I went […] When I started explaining to the doctor my problem, the translator was explaining a different thing that made me to be given Panadol [paracetamol] only. But after explaining without being translated, other drugs like amoxicillin, tests, and so many others were added” (participant 5, Madi FGD A)*.*

Difficulty translating undoubtedly contributed to the mismatch between what type of care refugees said they expected versus what they received. Almost uniformly, however, health and programme staff interpreted patient expressions of dissatisfaction as cultural, as well. For example, refugees’ preference for medicine delivered as an injection rather than oral tablets was perceived to be ignorant. A staff member from Mungulla Health Centre told us: “if you give them tablets, believe me, by evening they are going to be back”. Negative characterisations of people from South Sudan as rude, impatient, ignorant and stubborn peppered the descriptions of refugees by staff. Health providers often seemed overwhelmed by refugee needs and ill-equipped to interpret refugee care-seeking as anything other than unruly, saying, for example: “you can never please them [refugees], however much you may try, you fail” (staff member at Adjumani hospital). Similarly, South Sudanese people said health staff see refugees as “enemies” (Madi FGD A) and “talk to people in a bad way, they quarrel” (participant 1, Dinka FGD B).

Such confrontations led many refugees to question whether their right to healthcare was less legitimate than the surrounding host population’s, going so far as to wonder if health staff’s tendency not to use diagnostic tests in healthcare interactions was deliberate. One participant complained, for example, that “there is a microscope for doing the work but it’s just there and they are not using it. Maybe laboratory equipment for testing all the sicknesses is there but they are intentionally not using them” (participant 1, Madi FGD A). Moreover, such tensions between providers and patients and among staff at facilities likely made it very difficult to have the types of conversations necessary to prompt use of RDTs and contribute data for strategic decision-making by the surveillance programme.

#### Reluctance to engage humanitarian structures

Sleeping sickness experts especially outside of Uganda believed that a lack of political will among humanitarian responders to participate in elimination might have been to blame for the apparent absence of activities for refugees. As stated by a WHO representative at an international meeting in 2016:

In a refugee situation the priority is not sleeping sickness; [mal]nutrition, cholera is there, it is difficult to convince the actors to put attention here. We are the ones who know we are risking something, that something needs to be done. So we are trying to move the NGOs and UNHCR to at least put this issue on the table*.*

Indeed, the facility-level communication issues outlined above reflect the chaotic circumstances, political sensitivities and rationing inherent to operating health services in a crisis context.

However, it’s unlikely that humanitarian health workers and coordinators were opposed to participating in a sleeping sickness response. As described by staff from Ayilo Health Centre who told us, “we haven’t yet got microscopes, our laboratory is not fully ready but we have rapid tests”, many humanitarians viewed their access to RDTs for other diseases as a useful short-cut to having full lab infrastructure. Likewise, refugee groups we spoke with also unanimously wanted access to a greater variety of all sorts of blood tests. Rather, such RDTs for sleeping sickness were often not made available to responders through the integrated structures they were supposed to be working in.

How humanitarian actors typically engage vertical programmes in Uganda was described to us by a UNHCR health advisor this way: “when it comes to vertical programmes like tuberculosis, UNHCR doesn’t buy the drugs, the district does and the quality assurance. UNHCR programmes just provide the service and dispense.” With sleeping sickness RDTs no longer part of the standard tests available within government systems in Adjumani and southern Arua, however, sleeping sickness did not feature in discussions between humanitarian actors and other district government authorities in charge of general refugee health integration. Consequently, most humanitarian practitioners we spoke to associated sleeping sickness only with the previous refugee crisis in Uganda and were unaware of any elimination efforts. Moreover, outside of sentinel surveillance facilities, no UNHCR, NGO manager or facility staff we spoke with in 2015 were aware that they had responsibilities to refer syndromic suspects to sentinel detection sites, reflecting a lack of communication between the ISSEP and humanitarian actors at a local and regional level.

ISSEP coordinators, themselves, admitted they were reluctant to engage in communication with responders. Partly this reluctance related to their belief that any activities unplanned in the ISSEP design, such as the comprehensive screening of refugees desired by COCTU authorities, were deemed to require further funding by international donors. As one coordinator argued in 2014, “1 case of sleeping sickness is worth 600 cases of malaria in terms of management” and “targeting all of them [settlements] requires a lot of resources, it takes time to raise these”. More fundamentally, however, for all district supervisors and national coordinators interviewed between 2014 and 2016, refugees were perceived not to be the responsibility of the national sleeping sickness programme, leaving a clear gap in sleeping sickness elimination governance. According to a former international sleeping sickness programme manager, this is an attitude that has evidently not changed since the last humanitarian crisis in early 2000, despite national initiatives to improve cross-sectoral coordination of trypanosomiasis activities through the UTCC and COCTU [14].

## Discussion

This study has demonstrated an important limitation to Uganda’s integrated refugee policy by observing the national sleeping sickness programme’s response to an influx of South Sudanese refugees from 2013 to 2016. We observed several entrenched norms and practices that worked against integration of refugees into the national sleeping sickness medical surveillance system, despite the availability of a promising technological innovation, an RDT, that could be deployed in the government-controlled spaces where refugees were being provided care.

Before the refugee influx, Adjumani District was assumed to have disease prevalence so low that there would be little need for surveillance. These assumptions were not contradicted by RDT-based monitoring data produced during the first six months of the programme, so the programme reduced surveillance intensity by removing RDTs from most facilities in the district. This had harmful, if unintended, consequences for surveillance equity in West Nile, as Adjumani was the place where most refugees were sent and sleeping sickness experts both within and outside the country believe refugees are at particular risk of disease.

This story has important implications for global sleeping sickness programmes seeking to uphold commitments to addressing disease in vulnerable populations as well as the legitimacy of their claims about elimination. Finding no cases from areas or populations that have been largely excluded from surveillance appears as a programmatic success but it may alternatively be explained as a failure of implementation. Ethnographic study of other global health programmes in Uganda [45] and elsewhere [46] suggest important incentives for coordinators not to “look beneath the surface” of successful outcomes data “to see how stated practice relate[s] to actual behaviour” because of the need to prove normative progress towards global goals or to justify subsequent rounds of funding [45]. Indeed, the need to prove that elimination was happening is a likely explanation for so many refugees’ social exclusion from surveillance in West Nile.

Poor quality implementation was anticipated here because of historical expectations ISSEP coordinators had about the quality of their relationships to Adjumani and some other districts. Additionally, district and national supervisors felt reluctant to engage with refugee-specific issues such as communicating with government health staff recruited for the humanitarian surge response to familiarise them with RDTs, their expected role in the elimination programme, or address their diagnosis-related communication challenges with patients. Coordinators also, however, wanted to demonstrate to donors that they were spending resources efficiently. So, in practice, staff not using RDTs at sufficient levels to justify the cost of monitoring was at least as important a reason for withdrawing RDTs from facilities as was coordinators’ perception of low sleeping sickness prevalence in the district. The absence of international guidance on the quality of surveillance required to produce evidence of elimination before restricting resources in a sentinel surveillance strategy also presumably contributed.

Fully integrating refugees into vertical health programmes coordinated at the national level seems to be a common problem in Uganda. This may not have affected sleeping sickness control until recently, however, because medical humanitarian agencies have historically been such central actors involved in sleeping sickness interventions in this region [14, 24]. Concerned with both the high mortality of this disease during epidemics and the affected populations who were displaced by conflict, there was substantial overlap in these agencies’ refugee health and sleeping sickness mandates and their responses typically occurred parallel to government structures. As disease has receded, however, humanitarians have disengaged with sleeping sickness control globally and endemic country governments keen to ‘accelerate’ progress towards elimination are now firmly in the driving seat in elimination programmes. It is perhaps not surprising then, that the ISSEP, which channels international funds through the Ministry of Health, has had difficulty integrating refugees. Refugees have not been the Ministry’s responsibility by long-standing tradition in sleeping sickness [14].

Forced displacement, however, is a growing problem globally. Notably, all 36 countries at risk of sleeping sickness host forcibly displaced populations including refugees, internally displaced persons or recently returned displaced people, with nearly half (17 or 47.2%) supporting large displaced populations of at least 50,000 people, many of whom could be living in areas which support transmission (Additional file 2). New norms, incentives or structures thus urgently need to be established to ensure the needs of displaced people are not left behind by government sleeping sickness programmes in their enthusiasm to demonstrate elimination progress. The Global Fund to Fight AIDS, Tuberculosis and Malaria has successfully encouraged integration in some national malaria elimination programmes by prompting countries to include additional provisions for refugees in their applications [4]. The SDGs commit countries to monitoring progress towards all targets according to characteristics of vulnerable populations, including migratory status [47]. Elimination programmes can also be evaluated on their inclusiveness towards forced migrant populations such as whether health outcomes for both host and migrant populations are improved, something which was beyond the scope of our investigations [20].

This study also highlighted important limitations of RDTs to produce quality and relevant data for elimination. Despite the technology’s appealing simplicity, RDTs are always controlled by human decision-making and behaviour. This is especially important for sleeping sickness programmes like the ISSEP which have abandoned more systematic approaches to case detection, and rely instead on social interactions between patients and providers to identify syndromic suspects for testing. Refugees in West Nile had to overcome substantial communication challenges, sometimes including discrimination, to leave a health consultation feeling satisfied. In other settings in Africa, people who have difficulty communicating their health problems to staff because of differences in literacy, class or ethnicity typically come away with fewer medicines [48]. Crossing an international border also sharply affects power relations even when refugees and the host population belong to the same ethnic group [49]. We should assume that it is more difficult for a national health worker to suspect that a refugee than a citizen patient is affected by sleeping sickness through conversations about symptoms and alternative diagnoses, given how exasperated with service delivery both parties felt. While health providers may not like refugees standing over them, demanding to know what tests and medicines they are prescribing, the sleeping sickness literature consistently concludes that patients need to be persistent seekers of healthcare in order to receive a correct diagnosis [17, 50]. Indeed, patient-led detection is a key reason why internally displaced people have been detected so successfully elsewhere [17]. Persistence in healthcare should thus not be dismissed as bad behaviour in the refugee context.

### Recommendations

Despite their structural and historical basis, the problems highlighted above can be overcome in West Nile and avoided elsewhere. Donor conditionality that incentivises programmes to anticipate refugee needs and disaggregate reporting on vulnerable groups could improve programmes’ relationships with facilities that serve refugees. National trypanosomiasis coordinating bodies (such as COCTU in Uganda) can also promote the integration of migrant populations in national NTD programme policies, budgets and plans, per states’ commitments to the SDGs. How best to overcome systemic integration issues such as communication between government and humanitarian structures should be considered, to ensure that diagnostics are available and used in government and private facilities serving refugees who are at risk of disease. These spaces are key for the successful implementation of both elimination programmes and refugee integration policies. At RDT trainings for health staff, persistence in health-seeking as positive patient behaviour in sleeping sickness could be discussed and translators and community liaisons could also be invited to increase staff and patients’ awareness of available sleeping sickness diagnostics. International guidance on how long and at what intensity surveillance diagnostics should be kept in place is an outstanding issue [26]. In the interim, supervisors and coordinators should be encouraged to investigate operational reasons for very low use of RDTs in individual facilities.

## Conclusions

Monitoring equity between forcibly-displaced and host populations will be a key challenge for disease elimination programmes in the SDG era. Despite a promising policy context for the integration of South Sudanese refugees into national medical surveillance systems for sleeping sickness elimination in Uganda, we observed key governance and operational challenges that limited refugees’ equitable inclusion. These ranged from perceived donor pressure to contain costs, to local and inter-sectoral coordination issues, to cross-cultural communication challenges with using RDTs. Unclear international guidance on how to use RDTs as a new technology in an elimination context also contributed. Though they have a historical basis, these challenges can be overcome. We support recent calls for a more robust, internationally-supported but government-led response that specifically meets the needs of refugees living in Uganda and uses all available tools [42]. Elimination programmes which marginalise forced migrants risk unwittingly contributing to disease spread [1, 46] and reinforce social inequities, so new norms need to be established at local, national and international levels.

## Additional files

Additional file 1: Identifying populations at most risk. Additional detail on the method used to identify the refugee populations in West Nile who were at most risk of being infected with sleeping sickness, including tables of cases reported from South Sudan and Uganda. (DOCX 27 kb)

Additional file 2: Forcibly-displaced persons living in sleeping sickness endemic countries. A table indicating numbers of sleeping sickness cases and forcibly displaced populations by country. (DOCX 22 kb)

## Abbreviations

COCTU: Coordinating Office for Control of Trypanosomiasis in Uganda
FGD: Focus group discussions
FIND: Foundation for Innovative New Diagnostics
HAT: Human African trypanosomiasis
IDP: Internally displaced person
ISSEP: Intensified Sleeping Sickness Elimination Project
LAMP: Loop-mediated isothermal amplification
LED: Light-emitting diode
NGO: Non-governmental organisation
NTD: Neglected tropical disease
OPM: Office of the Prime Minister
RDT: Rapid diagnostic test
SDG: Sustainable Development Goal
UNHCR: United Nations High Commissioner for Refugees (United Nations Refugee Agency)
UTCC: Uganda Trypanosomiasis Control Council
WHO: World Health Organisation

## Footnotes

1 Apart from this passive approach to case detection, western districts of the West Nile region have benefitted from tsetse control activities using insecticide-treated targets established under a 2011 pilot programme. Under the multi-country Trypa-No! project, Adjumani is expected to be included in 2017. Mobile team-based screening was also used on a small scale in the pilot to measure the project’s impact [27].

2 Major refugee settlements in West Nile were established in Adjumani and Arua Districts in 2014 and 2015 and in Yumbe and Amuru Districts in 2016. As of 30 Sep 2016, more than half of South Sudanese refugees in Uganda (58%) were living in settlements in Adjumani District [51].

3 Whereas pre-2016, most displacement to Uganda came from non-endemic areas of South Sudan, the increase in fighting in southern states since July 2016 means more refugees are now coming from disease-affected areas [52, 53] and they are settling inside and outside of Adjumani.

4 Based on experience with the CATT diagnostic test positioned in central facilities, recommended global strategy for areas of high transmission intensity is to reduce surveillance to a restricted number of ‘sentinel’ sites once no cases have been reported from an area in the last five years, during which time control measures such as outreach-based screening should have been in place [26].
