# Supplementary material for: Including refugees in disease elimination: challenges observed from a sleeping sickness programme in Uganda
Source: Confl Health. 2017 Dec 1;11:22. doi: 10.1186/s13031-017-0125-x (PMC5710113; doi:10.1186/s13031-017-0125-x)
Supplement: Supplementary file 1 — Identifying populations at most risk. Additional detail on the method used to identify the refugee populations in West Nile who were at most risk of being infected with sleeping sickness, including tables of cases reported from South Sudan and Uganda. (DOCX 27 kb) [file 13031_2017_125_MOESM1_ESM.docx]

# Additional file 1: Identifying populations at most risk

70% of refugees and asylum-seekers living in Uganda today originate from South Sudan, and Uganda is the largest receiving country for South Sudanese refugees [1].

The potential burden of sleeping sickness in South Sudanese refugees is unknown. As sleeping sickness is an increasingly rare disease (typically affecting only around 5% of a population in an emergency outbreak and prevalent at <1% in most endemic areas today), ascertaining public health need for a potentially costly sleeping sickness response is always uncertain [2]. Additionally, public knowledge, memory and perceptions of sleeping sickness are known to be highly geographically-specific, reflecting the focal epidemiology of the disease which can affect some villages for generations but leave neighbouring villages untouched [3, 4].

At the time of research, no systematic, mobile team-based screening for sleeping sickness had taken place in refugee populations and facility-based surveillance data based on passive screening of refugees served at Ugandan facilities was complex to interpret. Historical prevalence and risk maps generated by the World Health Organisation [5], and screening and case data provided by national programmes (see Tables 1 and 2) offered some insight into whether refugee populations might have carried sleeping sickness from endemic areas or been at risk of infection in areas they settled. While many refugees had settled across West Nile which harbours tsetse flies capable of transmitting the disease, the majority of refugees came from areas not endemic for sleeping sickness. We targeted our research enquiries to West Nile refugee populations in which ongoing sleeping sickness transmission was most likely to be occurring, based on the endemicity of sleeping sickness in their areas of origin or migration routes.

Table 1. Coverage of sleeping sickness RDT-based integrated facility-based screening programmes in Uganda and South Sudan

|  | **Time period RDTs available** | **% at-risk population covered (at peak)** | **Population covered*** | **# Facilities with RDTs available (at peak)** | **# People screened with RDTs** | **% RDT-positive referrals completed** | **# sleeping sickness cases identified** | **Prevalence among people screened**** |
| --- | --- | --- | --- | --- | --- | --- | --- | --- |
| Uganda | Sep 2013 - Aug 2016 | 100 | 2.2 million | 212 | 19,134 | 77 | 17 | 0.1% |
| South Sudan | Jul 2015 - Jun 2016 | 58 | 1.1 million | 135 | 10,609 | 30 | 16 | 0.5% |

*See maps in manuscript Figure 1 and [6] for geographic programme coverage. Screening with standard diagnostics was otherwise available at a single hospital in most other disease foci of South Sudan. Since the increase in fighting within southern areas of the country in July 2016, however, most sleeping sickness activities in South Sudan have been suspended. **Assuming that the true prevalence is similar among RDT-positive patients who did and did not complete their referral. More active methods of control (such as mobile team-based systematic screening and/or tsetse control) are typically recommended when the population prevalence of sleeping sickness exceeds 1%. Source: Data come from five presentations given at the 2016 Joint EANETT-HAT Platform scientific and steering committee meetings, “Elimination of HAT: a same objective for both research and control”, in Conakry, Guinea, 19-22 Sep 2016. Presenters included C Wamboga, O Erphas, A Chol, R Poni and A Mumbo.

Table 2. Number of sleeping sickness cases reported and people screened in South Sudan in 2015, by disease focus

| **Disease focus** | **People screened** | **Cases detected** |
| --- | --- | --- |
| Nimule | 148 | 12 |
| Juba | 62 | 1 |
| Yei | 604 | 18 |
| Lui | 146 | 10 |
| Yambio | 86 | 4 |
| Tambura | 34 | 0 |
| Total | 1080 | 45 |

Legend: Reproduced with permission from [7]

UNHCR collects information on refugees’ area of origin routinely when refugees register, however, information linking peoples’ area of origin with their current place of residence is not routinely analysed and available to the public. It also appears to be not easily available to staff working in regional or capital offices of UNHCR, the Office of the Prime Minister (OPM) which oversees refugee affairs in Uganda, or partner non-governmental organisations (NGOs) which implement services. We therefore relied on UNHCR, OPM and NGO staff’s knowledge of refugee ethnicity predominating in different settlements and the knowledge of refugee leaders within these settlements to identify populations coming from specific areas of South Sudan to focus our enquiries.

Dinkas were the largest ethnic group, representing half of South Sudanese refugees in Uganda followed by Nuers who represented a fifth [8]. Many Dinka people came from Bor, Juba and Nimule. While Juba is a minor historic focus and the transmission potential in Bor is unclear, a minimum of 10 cases of sleeping sickness per year have been detected from the Nimule/Magwi focus alone, since 2005 (personal communication, Elizeous Surur in 2016). Dinka people have been displaced in Nimule since 1991 but in the first few months of the current crisis many more Dinka sought refuge in the Nimule area and Nimule was located on an important migration route to Uganda. Of 35,000 Dinka people registered with the South Sudan Relief and Rehabilitation Commission between December 2013 and March 2014, only 10,000 remained in Nimule, with most continuing on to Uganda [9]. Subsequent violence in the Nimule area in December 2014 and July 2015 also prompted migration from both remaining Dinkas and ethnic Madis who historically live there [10, 11]. Residents from Nimule could be found in Ayilo and Nyumanzi settlements (Dinka refugees) and the Maaji settlements (Madi refugees). All were located in Adjumani District which is endemic for sleeping sickness [5, 12]. We focused our research in these settlements in 2015 which had a combined population of 58,719 (data shared by UNHCR). With the exception of populations from Juba, most Nuer refugees had migrated from areas non-endemic for sleeping sickness and settled in non-endemic areas outside of West Nile (mainly Kiryandongo and Kampala) [8].

Since the increase in fighting within South Sudan in July 2016, more refugees are now coming from other ethnic groups from the southern-most states which are disease-affected, particularly Yei/Kajo-Keji [5, 13].

**References cited:**

1. UNHCR. *Uganda: 2016 Year end report*. 2017 [cited 24/7/2016] Available from: <http://reporting.unhcr.org/node/5129>.

2. WHO, Control and surveillance of human African trypanosomiasis: report of a WHO expert committee. 2013, World Health Organisation Available from: <http://apps.who.int/iris/bitstream/10665/95732/1/9789241209847_eng.pdf?ua=1>.

3. Kovacic, V., et al., *We Remember... Elders' Memories and Perceptions of Sleeping Sickness Control Interventions in West Nile, Uganda.* PLoS Negl Trop Dis, 2016. **10**(6): p. e0004745.

4. Kovacic, V., et al., *Community acceptance of tsetse control baits: a qualitative study in Arua District, North West Uganda.* PLoS Negl Trop Dis, 2013. **7**(12): p. e2579.

5. WHO. *Mapping the distribution of human African trypanosomiasis*. Undated [cited 12/8/2016] Available from: <http://www.who.int/trypanosomiasis_african/country/foci_AFRO/en/>.

6. FIND. *Project update South Sudan: Integrating and intensifying control of sleeping sickness in the primary health-care system*. 2016 [cited 5/12/2016] Available from: <http://www.finddx.org/wp-content/uploads/2016/09/HAT-SouthSudan-WEB-Aug2016.pdf>.

7. Alak, A.C.D., *South Sudan country presentation*, in *4th Joint Scientific Meeting of the HAT Platform-EANETT*. 2016: Conakry.

8. Ambroso, G., et al., Evaluation of UNHCR’s response to the L3 South Sudan refugee crisis in Uganda and Ethiopia. 2016, UNHCR [cited 4/8/2016] Available from: <http://reliefweb.int/report/ethiopia/evaluation-unhcrs-response-l3-south-sudan-refugee-crisis-uganda-and-ethiopia>.

9. Sudan Tribune, S. Sudan leaders agree to allow IDPs to remain in Nimule amid local protests. 2014, [cited 16/8/2016] Available from: <http://www.sudantribune.com/spip.php?article50314>.

10. Sudan Tribune, S. Sudan army says military settlements uncovered in Eastern Equatoria. 2014, [cited 16/8/2016] Available from: <http://www.sudantribune.com/spip.php?article53424>.

11. Sudan Tribune, South Sudanese rebels warn civilians to evacuate from border town. 2015, [cited 16/8/2016] Available from: <http://www.sudantribune.com/spip.php?article55640>.

12. UNHCR, South Sudan refugee emergency response July 2016 (Uganda). 2016, UN Refugee Agency [cited 5/12/2016] Available from: data.unhcr.org/SouthSudan/download.php?id=2982.

13. UNHCR, South Sudan emergency: Areas of origin (states) of South Sudanese refugees in Uganda. 2014, UN Refugee Agency [cited 5/12/2016] Available from: data2.unhcr.org/fr/documents/download/29714.
