# Supplementary material for: Including refugees in disease elimination: challenges observed from a sleeping sickness programme in Uganda
Source: Confl Health. 2017 Dec 1;11:22. doi: 10.1186/s13031-017-0125-x (PMC5710113; doi:10.1186/s13031-017-0125-x)
Supplement: Supplementary file 2 — Forcibly-displaced persons living in sleeping sickness endemic countries. A table indicating numbers of sleeping sickness cases and forcibly displaced populations by country. (DOCX 22 kb) [file 13031_2017_125_MOESM2_ESM.docx]

# Additional file 2: Forcibly-displaced persons living in sleeping sickness endemic countries

|  | **Sleeping sickness cases in 2014** | **Refugees & people in refugee-like situations in 2014** | **Returned refugees in 2014** | **Internally displaced persons (IDPs) in 2014** | **Returned IDPs in 2014** | **Total population forcibly displaced** |
| --- | --- | --- | --- | --- | --- | --- |
| **Countries at risk of *Tb gambiense* sleeping sickness** | | | | | | |
| Angola | 35 | 15,474 | 14,284 |  |  | 29,758 |
| Cameroon | 7 | 264,126 | 385 | 40,000 |  | 304,511 |
| Central African Republic | 194 | 7,694 |  | 438,500 |  | 446,194 |
| Chad | 95 | 452,897 | 370 | 71,000 |  | 524,267 |
| Congo | 21 | 54,842 | 14 | 7,800 |  | 62,656 |
| Cote d'Ivoire | 6 | 1,925 | 12,362 | 300,900 | 5,000 | 320,187 |
| Democratic Republic of Congo | 3,206 | 119,754 | 25,150 | 2,756,600 | 561,100 | 3,462,604 |
| Equatorial Guinea | 0 | 174 |  |  |  | 174 |
| Gabon | 10 | 1,013 |  |  |  | 1,013 |
| Guinea | 33 | 8,766 |  |  |  | 8,766 |
| Nigeria | 0 | 1,239 |  | 1,075,300 |  | 1,076,539 |
| Sierra Leone | No data | 1,372 |  |  |  | 1,372 |
| South Sudan | 63 | 248,152 |  | 1,498,200 | 206,000 | 1,952,352 |
| Uganda | 9 | 385,513 | 1 | 29,800 |  | 415,314 |
| **Countries at marginal risk of *Tb gambiense* sleeping sickness** | | | | | | |
| Benin | 0 | 415 |  |  |  | 415 |
| Burkina Faso | 0 | 31,894 |  |  |  | 31,894 |
| Gambia | No data | 11,608 |  |  |  | 11,608 |
| Ghana | 0 | 18,450 |  |  |  | 18,450 |
| Guinea-Bissau | No data | 8,684 |  |  |  | 8,684 |
| Liberia | No data | 38,595 |  | 23,000 |  | 61,595 |
| Mali | 0 | 15,195 | 20,961 | 61,600 | 178,400 | 276,156 |
| Niger | No data | 77,830 |  | 11,000 |  | 88,830 |
| Senegal | No data | 14,274 |  | 24,000 |  | 38,274 |
| Togo | 0 | 21,778 | 28 | 10,000 |  | 31,806 |
| **Countries at risk of *Tb rhodesiense* sleeping sickness** | | | | | | |
| Burundi | No data | 52,936 | 1,350 | 77,600 | 1,300 | 133,186 |
| Kenya | 0 | 551,352 | 1 | 309,200 |  | 860,553 |
| Malawi | 32 | 5,874 |  |  |  | 5,874 |
| Mozambique | No data | 4,536 |  |  |  | 4,536 |
| Uganda | 70 |  |  |  |  |  |
| United Republic of Tanzania | 1 | 88,492 |  |  |  | 88,492 |
| Zambia | 12 | 25,578 |  |  |  | 25,578 |
| Zimbabwe | 3 | 6079 | 55 | 36,000 |  | 42,134 |
| **Countries at marginal risk of *Tb rhodesiense* sleeping sickness** | | | | | | |
| Botswana | No data | 2,645 |  |  |  | 2,645 |
| Ethiopia | No data | 659,524 | 466 | 397,241 | 123,499 | 1,180,730 |
| Namibia | No data | 1,767 |  |  |  | 1,767 |
| Rwanda | No data | 73,820 | 5,787 |  |  | 79,607 |
| Swaziland | No data | 515 |  |  |  | 515 |

Legend: Endemic areas do not always overlap with refugee-hosting areas within countries. People in refugee-like situations: This category is descriptive in nature and includes groups of persons who are outside their country or territory of origin and who face protection risks similar to those of refugees, but for whom refugee status has, for practical or other reasons, not been ascertained. Internally displaced person (IDP): A person displaced within his/her country because of armed conflict, situations of generalised violence or violations of human rights. Returned refugees or IDPs: Refugees or IDPs who have returned to their place of origin within the last year.

Sources: Data on cases of sleeping sickness came from [1] and [2]; classifications of countries at risk or marginal risk followed [3]. Data on refugees came from [4] and on IDPs from [5]. Data on refugees in Equatorial Guinea came from [6] as this was unavailable in [4].

**References cited:**

1. WHO. *Number of new reported cases (T.b. gambiense): Data by country* 2016 [cited 17/11/2016] Available from: <http://apps.who.int/gho/data/node.main.A1636?lang=en>.

2. WHO. *Number of new reported cases (T.b. rhodesiense): Data by country*. 2016 [cited 17/11/2016] Available from: <http://apps.who.int/gho/data/node.main.A1637?lang=en>.

3. Simarro, P.P., et al., *Estimating and mapping the population at risk of sleeping sickness.* PLoS Negl Trop Dis, 2012. **6**(10): p. e1859.

4. UNHCR, UNHCR global trends in forced displaced in 2014. 2015, UN Refugee Agency Available from: <http://www.unhcr.org/uk/statistics/country/556725e69/unhcr-global-trends-2014.html>.

5. IDMC. *Global figures: Latest IDP numbers by country*. 2016 [cited 17/11/2016] Available from: <http://www.internal-displacement.org/global-figures/>.

6. Index Mundi. *Equatorial Guinea: Refugee population by country or territory of origin*. [cited 17/11/2016] Available from: <http://www.indexmundi.com/facts/equatorial-guinea/refugee-population-by-country-or-territory-of-origin>.
